# High-resolution PET detectors with dual-ended readout using cost-effective highly multiplexed readout circuit and MPT2321 ASIC

**DOI:** 10.1186/s40658-026-00908-x

**Published:** 2026-06-15

**Authors:** Zhiqiang Zhu, Denghong Ren, Weiming Chen, Wei Shen, Xianchao Huang, Ning Ren, Zhonghua Kuang, Zhanli Hu, Yongfeng Yang, Zheng Liu

**Affiliations:** 1 Changsha University of Science & Technology, Changsha, China; 2 National innovation center for advanced medical devices, Shenzhen, China; 3 Paul C. Lauterbur Research Center for Biomedical Imaging, Shenzhen Institute of Advanced d Technology, Chinese Academy of Sciences, No. 1068 Xueyuan Avenue, Xili, Nanshan District, Shenzhen, China; 4 MicroParity Technology, Hangzhou, China; 5 Beijing Engineering Research Centre of Radiographic Techniques and Equipment, Institute of High Energy Physics, Chinese Academy of Sciences, Beijing, 100049 People’s Republic of China

**Keywords:** PET detector, LYSO crystal, Silicon photomultiplier, ASIC, PET electronics

## Abstract

**Background:**

Small-animal positron emission tomography (PET) imaging is commonly employed in biomedical research to accelerate the translation of the discovery in the fields of molecular and cellular biology into the clinical practice. Its performance and cost are largely dependent on the employed detectors and electronics. In this work, a new electronics scheme aiming to simplify PET electronics is evaluated using two dual-ended readout PET detectors with high spatial resolution.

**Methods:**

The first detector is composed of a 16 × 16 lutetium–yttrium oxyorthosilicate (LYSO) array with crystal dimensions of 1.45 × 1.45 × 20 mm³. The second detector is composed of a 26 × 26 LYSO array with crystal dimensions of 0.75 × 0.75 × 20 mm³. Both LYSO arrays are dual-ended read out by 8 × 8 Hamamatsu S14161-3050HS-08 silicon photomultiplier (SiPM) arrays with a sensitive pixel area of 3 × 3 mm^2^ and a pitch of 3.2 mm. The 64 outputs of the SiPM array are read out by a highly multiplexed row–column summation network to convert them to four position-encoded energy signals. The four signals are processed by a commercial 32-channel MPT2321 ASIC. The flood map, energy resolution, depth of interaction (DOI) resolution, and timing resolution of the detectors are measured.

**Results:**

The first detector provided a flood map with all crystals resolved and an average peak-to-valley ratio (PVR) of 3.59 was obtained for the flood map quality, an energy resolution of 14.2 ± 0.4%, a DOI resolution of 3.04 ± 0.37 mm and a detector timing resolution (DTR) of 2.24 ± 0.10 ns. The second detector provided a flood map with all crystals resolved and an average PVR of 2.61 was obtained, an energy resolution of 15.3 ± 0.7%, a DOI resolution of 2.56 ± 0.46 mm and a DTR of 2.38 ± 0.20 ns.

**Conclusion:**

The electronics scheme evaluated in this study provides competitive energy and DOI performance for high-resolution small-animal PET detectors while significantly reducing electronics channel count and system complexity.

## Background

Preclinical small-animal imaging has played a central role in accelerating the translation of discovery in the fields of molecular and cellular biology into diagnostics and therapeutics that can be used in the clinical settings to prevent, diagnose, and treat diseases. Small-animal PET is commonly employed in oncological, neurological, and cardiovascular imaging owing to its highly sensitive and specific molecular imaging properties [1–4]. Compared with conventional clinical whole-body PET systems, small-animal PET systems require high-resolution and high-sensitivity performance because of the small organs sizes of the animals and the limitations with regard to the allowed injection dose for animals [5–8]. Thus far, advanced small-animal PET systems have achieved a spatial resolution of less than 1 mm and a center sensitivity of more than 10% [7, 9–13]. The goal of small-animal PET instrumentation research is to achieve one or more of the following features: high spatial resolution, high sensitivity, high timing resolution, and cost-effectiveness, which primarily depend on the detectors and electronics of the PET scanner.

The contribution of the photon acollinearity effect to the spatial resolution of a PET scanner with a detector diameter of D is 0.0022D mm [14]. In the widely used whole-body PET scanners with an approximately 800 mm detector ring diameter, the impact of photon acollinearity on spatial resolution is about 1.7 mm. In addition, TOF measurements mainly improve image signal-to-noise ratio in whole-body PET imaging because of the large imaging object size. Usually, detectors using a segmented scintillator crystal array, with crystals measuring 2–4 mm (width) × 20–30 mm (length) and single-ended readout by a SiPM array are used to overcome the trade-off between performance and cost of the scanner. Electronics with individual SiPM signal readouts are used to achieve a high timing resolution [15]. Generally, application-specific integrated circuits (ASICs) that can amplify and digitize the signals near the SiPMs are used in electronics to achieve a better timing resolution, achieve miniaturizations, and minimize the power consumption of the electronics.

In small-animal PET scanners featuring a 100–200 mm detector ring diameter, the impact of the photon acollinearity on spatial resolution is less than 0.5 mm. Moreover, the benefit of TOF measurements is limited owing to the relatively small imaging object size. Usually, detectors using a monolithic crystal of ~ 10 mm thickness that can attain ~ 1 mm spatial resolution, or a segmented crystal array with dimensions of 0.5–1.5 mm × 10–20 mm, are used to achieve a high spatial resolution. Highly multiplexed SiPM signal readout schemes such as resistor networks [16] or row-and-column summing [17] that degrade the detector timing resolution are used to minimize the number of readout channels. As detectors with a large crystal length-to-width ratio are commonly used in small-animal PET scanners, the spatial resolution is significantly degraded due to depth of interaction (DOI) uncertainty. Detectors with DOI measurement capability are preferred for high-resolution small-animal PET scanners [4, 18, 19]. Many DOI-measuring PET detector techniques, such as the single-ended readout of the segmented crystal array with DOI-dependent scintillation photon modulation [11, 20–22], dual-ended readouts of segmented crystal arrays [23–26], monolithic crystals [10, 27, 28] and semi-monolithic crystals [29–31], have been developed.

With the replacement of the PMTs by SiPMs and the increasing number of detectors in current PET scanners, the number of electronic channels in PET scanners has increased significantly. High-timing-resolution ASICs designed to process SiPM signals have been developed by both companies and research labs to satisfy the growing demand for PET electronics with a large number of channels [32–41]. For example, ASICs are being used in the newest whole-body and total-body PET scanners of Siemens [42, 43] and United Imaging [44, 45]. The 64-channel TOFPET2 ASICs developed by PETsys Electronics have been used by many groups worldwide in PET detectors and scanners [38]. Several generations of ASICs have been developed by Weeroc and tested for TOF PET detectors [40]. The 8-channel LUCAS ASIC was evaluated for TOF CT imaging [41]. Beyond the ASIC development, considerable efforts have focused on SiPM signal readout and multiplexing strategies to mitigate the rapidly increasing channel count. A comprehensive review by Park et al. [46] summarized the state-of-the-art SiPM signal readout and multiplexing techniques for PET systems, including both the analog and digital multiplexing approaches, and their trade-offs in timing resolution, energy resolution, and system complexity. More recently, Pagano et al. [47] evaluated multiplexed readout circuits combined with ASICs for brain PET scanner and conducted a cost-benefit analysis by directly comparing channel-reduced and non-reduced readout configurations. Their results provided important quantitative insights into the performance impact of channel reduction and offered practical guidance for the design of cost-effective next-generation PET systems.

Some studies have been performed to develop high-timing-resolution PET detectors with a multiplexed signal readout. Pourashraf et al. developed a 24:1 multiplexed timing readout circuit for their three-dimensional position-sensitive scintillation PET detectors and achieved a ~ 100 ps timing resolution for a detector employing 3 × 3 × 10 mm^3^ crystal elements with side readout [48]. Kang et al. developed a diode-based row–column multiplexing readout circuit (16:1) and achieved a timing resolution of 326 ps with the TOFPET2 ASICs for a detector with one-to-one crystal and SiPM coupling and a crystal dimensions of 3.1 × 3.1 × 20 mm^3^ [49]. A group led by Kyme also developed a row–column multiplexing readout circuit (9:1) and achieved a timing resolution of 1.3 ns with the TOFPET2 ASICs for a detector consisting of small dimensions of 0.785 × 0.785 × 20 mm^3^ [50]. PET detectors employing dual-ended readout with excellent timing performance were developed by using the NINO ASICs of CERN and the PICO2023 ASICs of Picotech SAS with a 4:1 timing multiplexing readout and separated timing and energy signal processing. A timing resolution of 142 ps was achieved for the detector using crystal dimensions of 1.45 × 1.45 × 20 mm^3^ with the PICO2023 ASICs [51].

The MPT2321 ASICs was recently developed by MicroParity Technology Inc., China, for the individual readout of the SiPM-based scintillator detectors with high timing precision [52, 53]. As TOF measurements using high-timing-resolution detectors are not required for small-animal PET scanners, an electronics scheme used for high-resolution PET detectors aimed at reducing the complexity of the PET electronics is proposed and evaluated in this work. The electronics scheme specifically consists of highly multiplexed signal readouts with the conventional row–column summation circuit and signal processing with the dedicated ASICs originally designed for individual SiPM signals. Two detectors consisting of a 16 × 16 LYSO array with a crystal dimension of 1.45 × 1.45 × 20 mm³ and a 26 × 26 LYSO array with a crystal dimension of 0.75 × 0.75 × 20 mm³ and dual-ended readout by 8 × 8 SiPM arrays were measured. This work demonstrates the viability of the proposed electronics scheme for the next-generation low cost and high performance small-animal PET systems since the number of electronics channels is reduced and the signal processing electronics is simplified by using the dedicated ASICs.

## Materials and methods

### Detector design

**Fig. 1:**
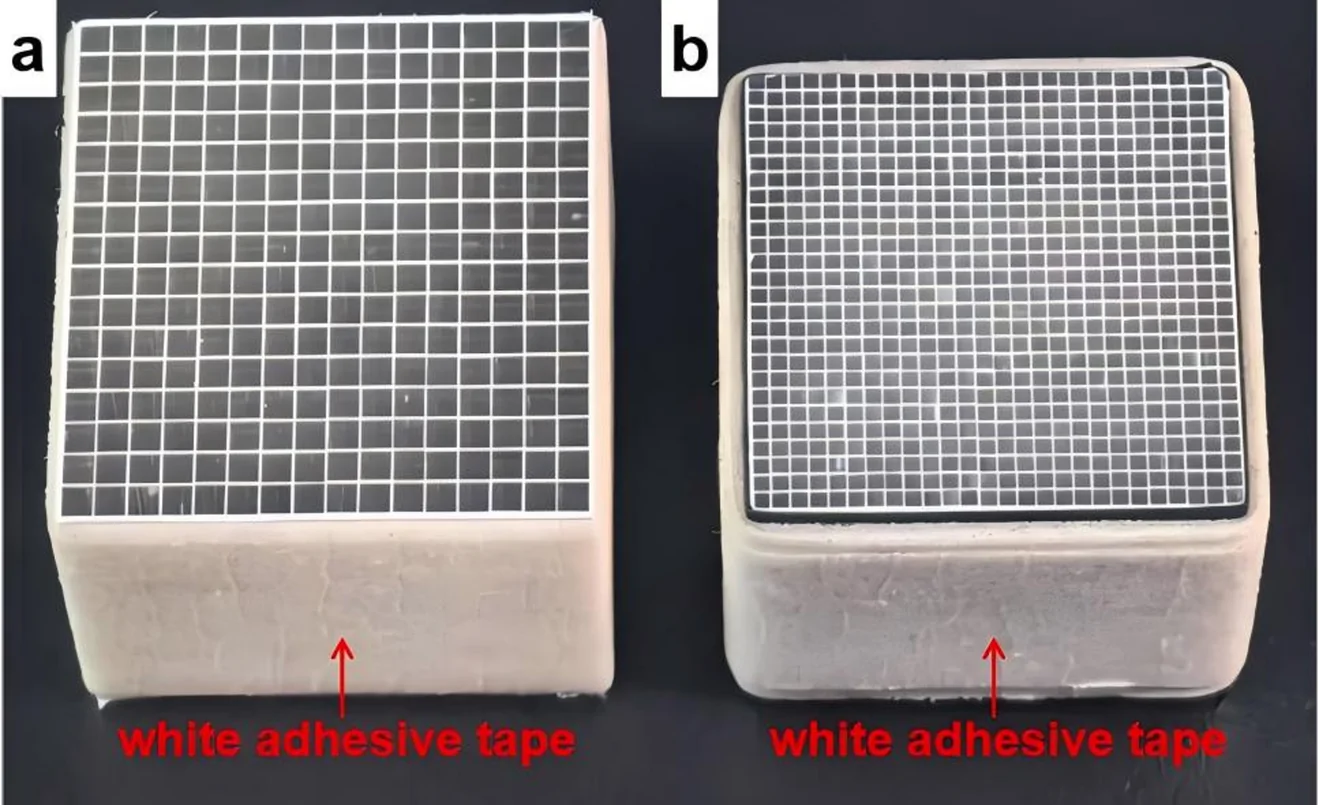
Photographs of (**a**) 16 × 16 LYSO array with crystal dimension of 1.45 × 1.45 × 20 mm³,  (**b**) 26 × 26 LYSO array with crystal dimension of 0.75 × 0.75 × 20 mm³

**Fig. 2:**
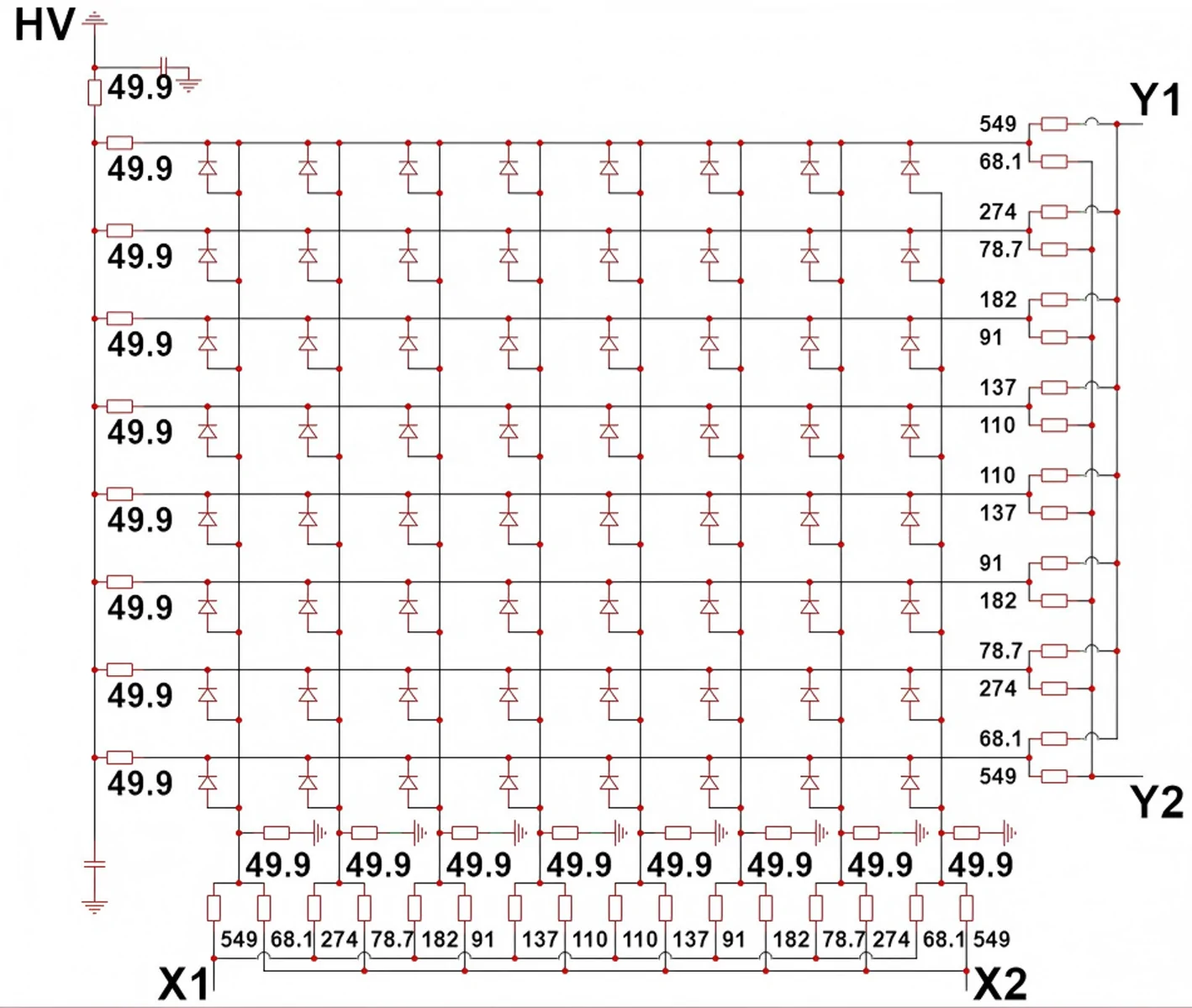
Schematic diagram of the signal multiplexing readout circuit of the 8 × 8 SiPM array

**Fig. 3:**
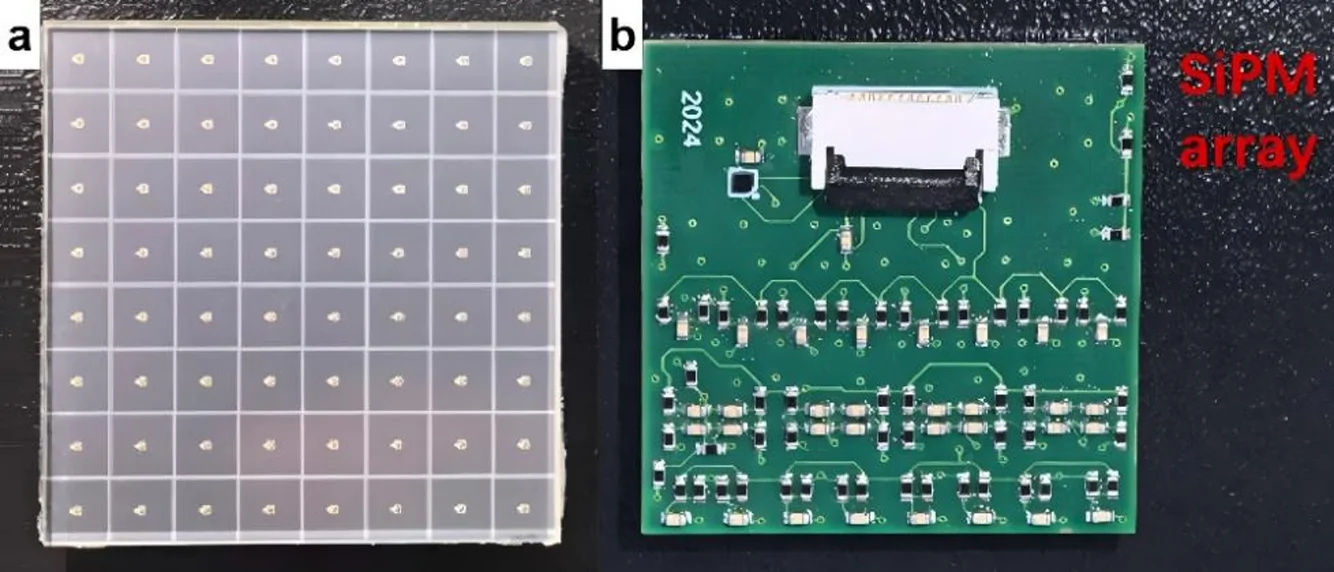
Photographs of (**a**) the 8 ⋅ 8 Hamamatsu SiPM array with an effective pixel area of 3 ⋅ 3 mm^2^ and a pixel pitch of 3.2 mm, (**b**) back of the signal readout circuit board of the SiPM array

**Fig. 4:**
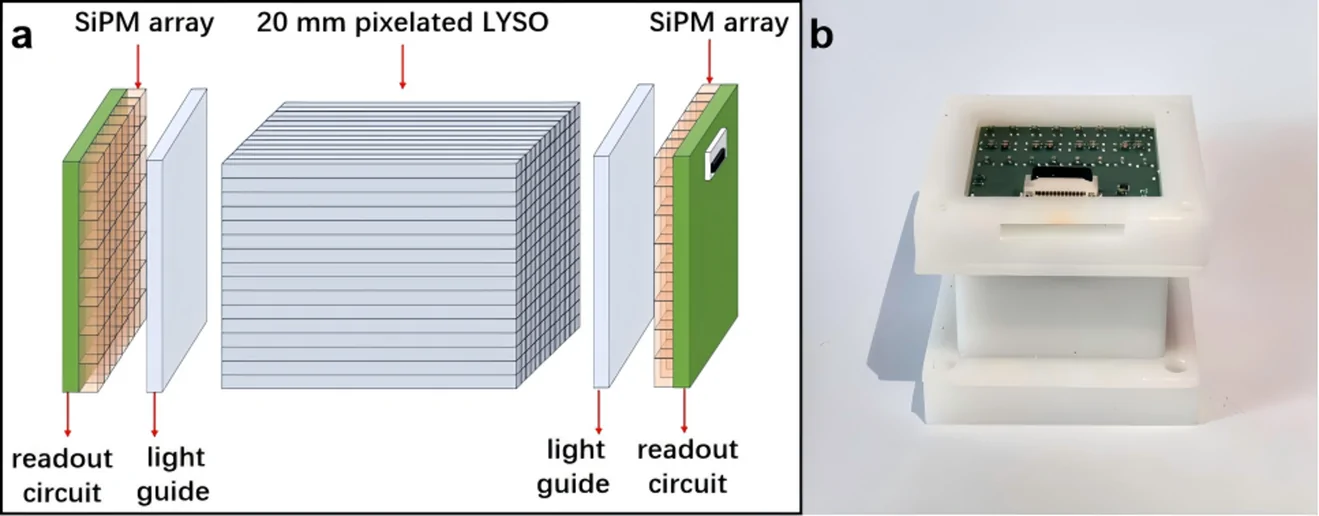
(**a**) Schematic and (**b**) photograph of the dual-ended readout PET detector

The two LYSO crystal arrays used in this work, as illustrated in Fig. 1, were fabricated by a commercial supplier (Ningbo EBO Optoelectronics Co. Ltd., Ningbo, China). All faces of the LYSO crystals were polished. The outer layer of the crystal array was wrapped with white adhesive tape to mechanically reinforce the crystals. The main features of the two crystal arrays are summarized in Table 1. The LYSO crystal array was read out from both ends by two 8 × 8 Hamamatsu S14161-3050HS-08 SiPM arrays, with each pixel having a sensitive area of 3 × 3 mm² and a 3.2 mm pitch. The multiplexing signal readout scheme of the SiPM array is similar to our previous work [54] and will be introduced here briefly. The anode signals from each row of the SiPMs are summed to yield eight row signals, while the cathode signals from each column are summed to yield eight column signals. The row and column signals are then combined with different weights through a resistive charge-division network to generate four position-encoding energy signals (X1, X2, Y1, and Y2). A schematic of the signal multiplexing readout circuit is shown in Fig. 2. Photographs of the 8 ⨯ 8 SiPM arrays and the signal-readout circuit of the SiPM array are illustrated in Fig. 3. A schematic and photo of a dual-ended readout PET detector is illustrated in Fig. 4. A 1 mm glass light guide was interposed between the crystal array and the SiPM array, and optical grease (Dow Corning XIAMETER) with an index of refraction of 1.4 was applied between both the crystal array and light guides as well as the light guides and SiPM arrays.

**Table 1:** Main features of the two crystal arrays

Detector #	Crystal Array	Crystal Dimensions (mm³)	Reflector/thickness (mm)
1	16 × 16	1.45 × 1.45 × 20	BaSO₄ /0.1
2	26 × 26	0.75 × 0.75 × 20	BaSO₄ /0.08

### The MPT2321 ASIC and signal processing electronics

**Fig. 5:**
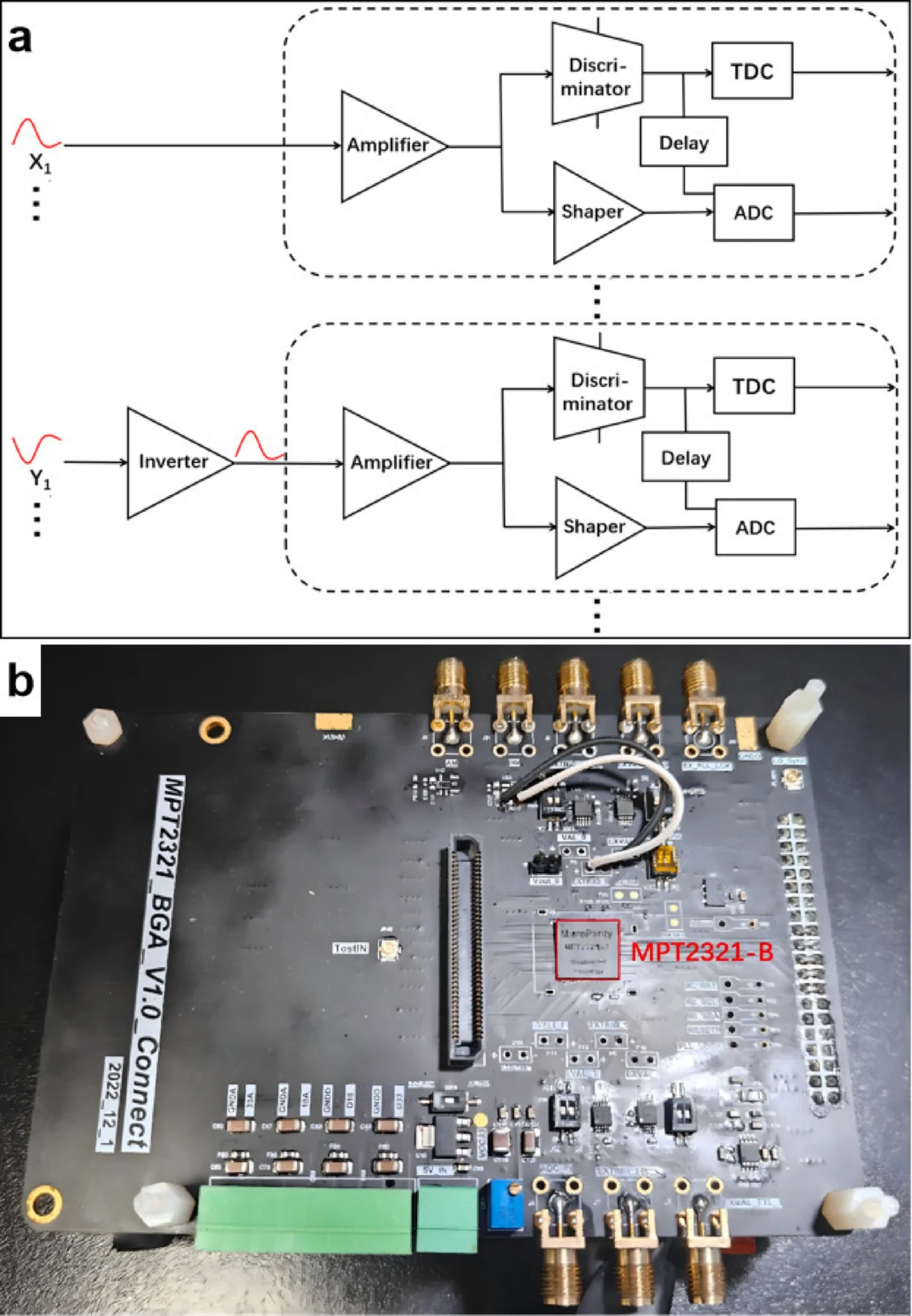
(**a**) Schematic of the signal-processing electronics, and (**b**) photograph of the electronics board based on the MPT2321 ASIC

The MPT2321 ASIC is a high-precision 32-channel chip developed by MicroParity Technology for SiPM-based detectors. The signal from the SiPM array is first amplified and then split into two, one for timing and the other for energy. The charge of one signal is integrated and the peak value is digitized by a 12-bit analog-to-digital converter (ADC). A trigger signal is generated using a comparator with a preset threshold using a leading-edge discrimination. The trigger signal with an appropriate delay is then used to determine the peak of the energy signal to be digitized by the ADC and is also digitized by a 20-bit time-to-digital converter (TDC) with a minimum TDC bin size of 100 ps to achieve a high-precision for the timing measurements. Although both positive and negative signals can be input to the MPT2321 ASIC, all 32 channels within a single chip must have the same polarity. In our setup, the positive X1 and X2 signals from the SiPM array are directly fed into the MPT2321 ASIC, while the negative Y1 and Y2 signals are inverted to positive polarity before input. For a full PET system employing multiple ASICs, signals of different polarities can be assigned to separate ASICs, eliminating the need for inversion. In all cases, the 64-to-4 multiplexing is performed using only passive components prior to the ASIC input. The threshold of the comparator and the delay of the trigger signal for peak detection can be preset and adjusted via software prior to data acquisition. The digitized data are transmitted through an LVDS high-speed serial interface and saved to a computer. A schematic of the signal-processing electronics and a photograph of the electronics circuit board based on the MPT2321 ASIC used in this work are shown in Fig. 5.

### Experimental setup and measurements

All measurements were conducted within a lightproof and temperature-stabilized box. The temperature within the enclosure was maintained at 16.0 ± 0.3 °C.

First, the best achievable timing resolution of the MPT2321 ASIC was evaluated using the coincidence of two identical detectors each composed of a 2 × 2 × 3 mm³ LYSO crystal coupled to a single SiPM with a 3 × 3 mm² active area. The two detectors were placed 15 mm apart, and a ^22^Na point source with a diameter of 0.3 mm and an activity of 352.61 kBq was positioned between the detectors. The signals from the two detectors were processed by two of the 32 channels of an MPT2321 ASIC chip. The timing difference between the two detectors was evaluated for different ASIC thresholds at an optimized SiPM bias voltage of 38 V.

**Fig. 6:**
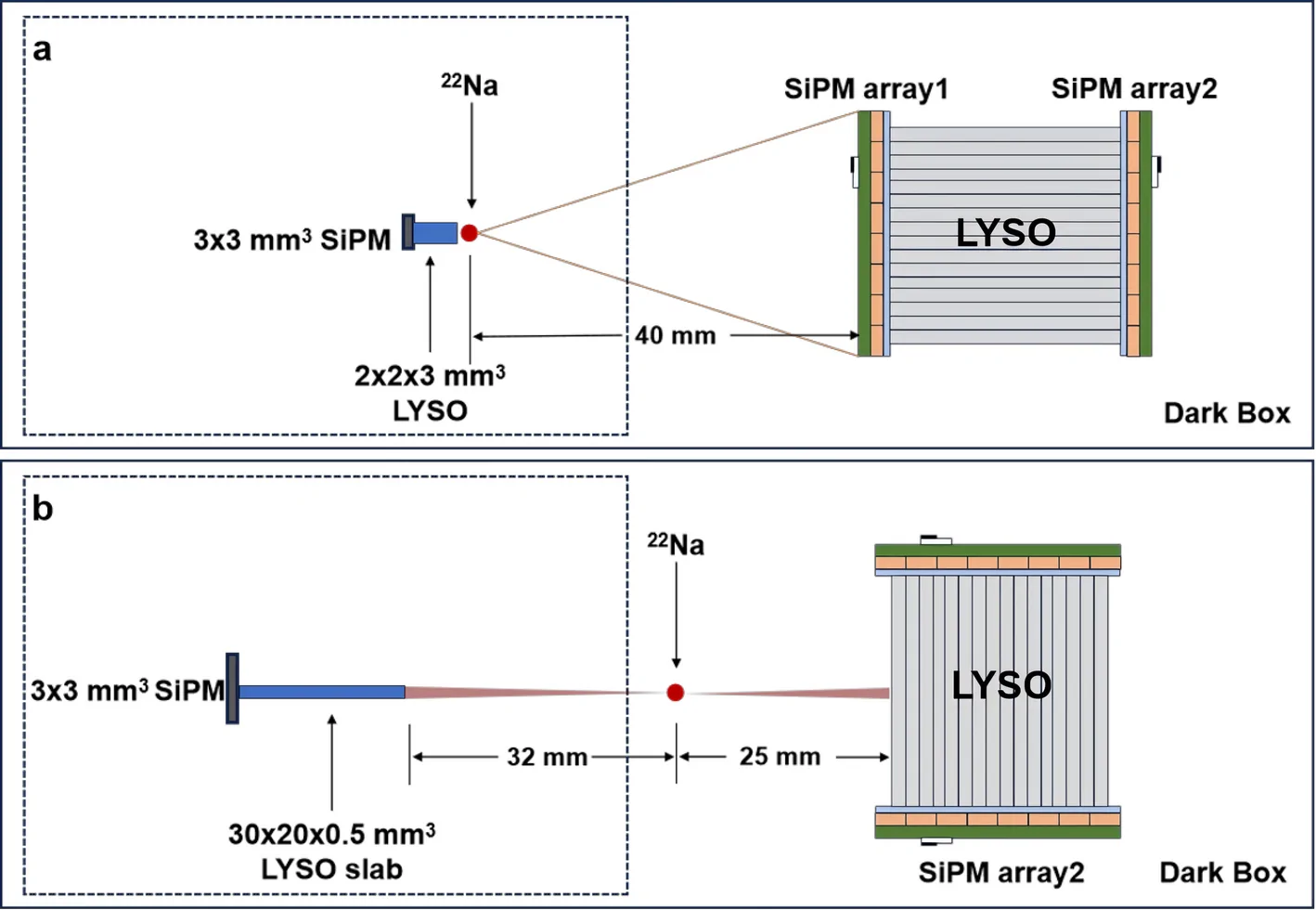
Experimental setups for measuring the (**a**) flood map, energy resolution and timing resolution using a reference detector composed of a single 2 × 2 × 3 mm³ LYSO crystal read out by a 3 × 3 mm² SiPM,  (**b**) DOI resolution using a reference detector composed of a 30 × 20 × 0.5 mm³ LYSO slab read out by a 1 × 8 SiPM array

Second, the flood histogram, energy resolution, and timing resolution of the two crystal arrays were evaluated using the experimental setup (Fig. 6a). The entire dual-ended readout detector was quasi-uniformly irradiated from the front by coincidence with a reference detector consisting of a 2 × 2 × 3 mm³ LYSO crystal coupled to a single SiPM with an active pixel area of 3 × 3 mm² and with the same ^22^Na point source placed close to the reference detector.

Third, the DOI resolution of the detector was measured using the experimental setup illustrated in Fig. 6b. The reference detector was composed of a 30 × 20 × 0.5 mm³ LYSO slab optically coupled to a 1 × 8 SiPM array with an active pixel area of 3 × 3 mm^2^. The same ^22^Na point source was positioned 32 mm and 25 mm from the reference and dual-ended readout detectors, respectively. Five depths of 2, 6, 10, 14, and 18 mm along the crystal array were irradiated using coincidence between the reference and the dual-ended readout detector. Both the reference detector and the point source were mounted on a translation stage. The bias voltage of the SiPM arrays for both PET detectors was set to 42.6 V. ~ 5 million events were acquired for the flood histogram, energy resolution and timing resolution measurements of both detectors and ~ 2 million events were acquired at each depth for the DOI resolution measurements of both detectors.

### Data analysis

The dual-ended PET detector produces eight position-encoding energy signals: X_t1_, X_t2_, Y_t1_, and Y_t2_ from the front SiPM array and X_b1_, X_b2_, Y_b1_, and Y_b2_ from the back SiPM array. The flood histogram coordinates (x, y) of an event are calculated using the equations below:

1 $$\:{\mathrm{x}}_{1}=\frac{{\mathrm{X}}_{\mathrm{t}1}}{{\mathrm{X}}_{\mathrm{t}1}+{\mathrm{X}}_{\mathrm{t}2}},\:\ {\mathrm{y}}_{1}=\frac{{\mathrm{Y}}_{\mathrm{t}1}}{{\mathrm{Y}}_{\mathrm{t}1}+{\mathrm{Y}}_{\mathrm{t}2}}$$

2 $$\:{\mathrm{x}}_{2}=\frac{{\mathrm{X}}_{\mathrm{b}1}}{{\mathrm{X}}_{\mathrm{b}1}+{\mathrm{X}}_{\mathrm{b}2}},\:\ {\mathrm{y}}_{2}=\frac{{\mathrm{Y}}_{\mathrm{b}1}}{{\mathrm{Y}}_{\mathrm{b}1}+{\mathrm{Y}}_{\mathrm{b}2}}$$

3 $$\:\mathrm{x}=\frac{{\mathrm{x}}_{1}+{\mathrm{x}}_{2}}{2},\:\:\mathrm{y}=\frac{{\mathrm{y}}_{1}+{\mathrm{y}}_{2}}{2}$$

To calculate the average flood map peak-to-valley ratio (PVR), the flood map is first segmented to generate a crystal lookup table that assigns regions to individual crystals. Specifically, the flood histogram was first smoothed to reduce statistical noise, and peaks of all individual crystals were identified using a local maximum detection method. The boundaries between adjacent crystals were then determined based on the midpoints between neighboring peaks, effectively partitioning the flood map into distinct crystal regions [55]. Thereafter, projection curves of each column (x) and row (y) of the crystals were extracted using all events assigned to each crystal. Peak values (P_i_, P_i+1_), which represent the peak heights of two adjacent crystals, and the valley value (Vi), which is the valley height between the two adjacent crystals, were obtained from the corresponding projection curves. The PVRs for adjacent crystal pairs along the x and y directions were calculated using Eqs. (4) and (5), respectively. For the detector performance evaluation reported in this study, only the PVR obtained from the line profile of the middle crystal row was used for quantitative comparison of flood histogram quality.

4 $$\:{\mathrm{P}\mathrm{V}\mathrm{R}}_{\mathrm{x}\mathrm{i}}=\frac{{\mathrm{p}}_{\mathrm{x}\mathrm{i}}+{\mathrm{p}}_{\mathrm{x}\mathrm{i}+1}}{2\cdot\:{\mathrm{V}}_{\mathrm{x}\mathrm{i}}}$$

5 $$\:{\mathrm{PVR}}_{\mathrm{yi}}\mathrm{=}\frac{{\mathrm{p}}_{\mathrm{yi}}\mathrm{+}{\mathrm{p}}_{\mathrm{yi+1}}}{2\cdot{\mathrm{V}}_{\mathrm{yi}}}$$

Where n is the row and column number of the crystal array. The average PVR of the detector is then obtained by averaging all PVR values calculated from both the x and y directions, as given by the equation below:

6 $$\:{\mathrm{P}\mathrm{V}\mathrm{R}}_{\mathrm{a}\mathrm{v}\mathrm{g}}=\frac{1}{2(\mathrm{n}-1)}(\sum\:_{\mathrm{i}=1}^{\mathrm{n}-1}{\mathrm{P}\mathrm{V}\mathrm{R}}_{\mathrm{x}\mathrm{i}}+\sum\:_{\mathrm{i}=1}^{n-1}{\mathrm{P}\mathrm{V}\mathrm{R}}_{\mathrm{y}\mathrm{i}})$$

Detector energy is calculated using the following equations:

7 $$\:{\mathrm{E}}_{1}={\mathrm{X}}_{\mathrm{t}1}+{\mathrm{X}}_{\mathrm{t}2}+{\mathrm{Y}}_{\mathrm{t}1}+{\mathrm{Y}}_{\mathrm{t}2}$$

8 $$\:{\mathrm{E}}_{2}={\mathrm{X}}_{\mathrm{b}1}+{\mathrm{X}}_{\mathrm{b}2}+{\mathrm{Y}}_{\mathrm{b}1}+{\mathrm{Y}}_{\mathrm{b}2}$$

9 $$\:\mathrm{E}={\mathrm{E}}_{1}+{\mathrm{E}}_{2}$$

Where E₁ and E₂ represent the energies detected by the front and back SiPM arrays, respectively. E denotes the combined energy detected by the two SiPM arrays. The energy spectrum for each crystal can be derived using the crystal lookup table. The 511 keV γ ray peak of an energy spectrum is then fitted with a Gaussian function, and the full width at half maximum (FWHM) divided by the photopeak amplitude is treated as the crystal energy resolution. The energy resolution of the detector is obtained by averaging the energy resolutions of all individual crystals. The measured photopeak amplitude of each crystal is also used to setup the energy window for event selection during the data analysis, which is 400–600 keV for all results of this work. The energy spectrum of the whole detector is also obtained after the crystal gain equalization using the measured photopeak amplitudes of individual crystals. The energy spectrum of the detector is fitted with a Gaussian function and the energy resolution of the whole detector is also obtained.

The DOI of the detector is derived from the energy ratio of the two SiPM arrays:

10 $$\:{\mathrm{D}\mathrm{O}\mathrm{I}}_{\mathrm{r}\mathrm{a}\mathrm{t}\mathrm{i}\mathrm{o}}=\frac{{\mathrm{E}}_{1}}{{\mathrm{E}}_{1}+{\mathrm{E}}_{2}}$$

The DOI ratio histograms of individual crystals at different depths can be obtained using the crystal lookup table of the detector. The DOI ratio histogram can be fitted with a Gaussian function, and the obtained FWHM DOI resolution can be expressed in millimeters through linear fitting of the DOI ratio peak positions and known depths of the crystal. The DOI resolution of a crystal can be obtained by an average of all 5 depths and the DOI resolution of the detector can be obtained by the value averaged over all crystals.

The average of the eight timings (the timing offset between the reference detector and each signal from the dual-ended readout detector) is used as the detector timing. The timing spectra of all individual crystals can also be obtained using the crystal lookup table. The FWHM of the timing spectrum (timing difference between the test and reference detectors) of a crystal is determined by a Gaussian fitting. The detector timing resolution (DTR) of a crystal in the test detector is calculated by quadratically subtracting the contribution of the reference detector. The DTR of the test detector is then obtained by averaging the DTR values over all crystals. The DTR of the reference detector was obtained from a measured CTR of approximately 296 ps FWHM between two identical reference detectors (DTR = CTR/√2) as shown in the Sect.  3.1.

## Results

### Timing resolution can be achieved with the MPT2321 ASIC

**Fig. 7:**
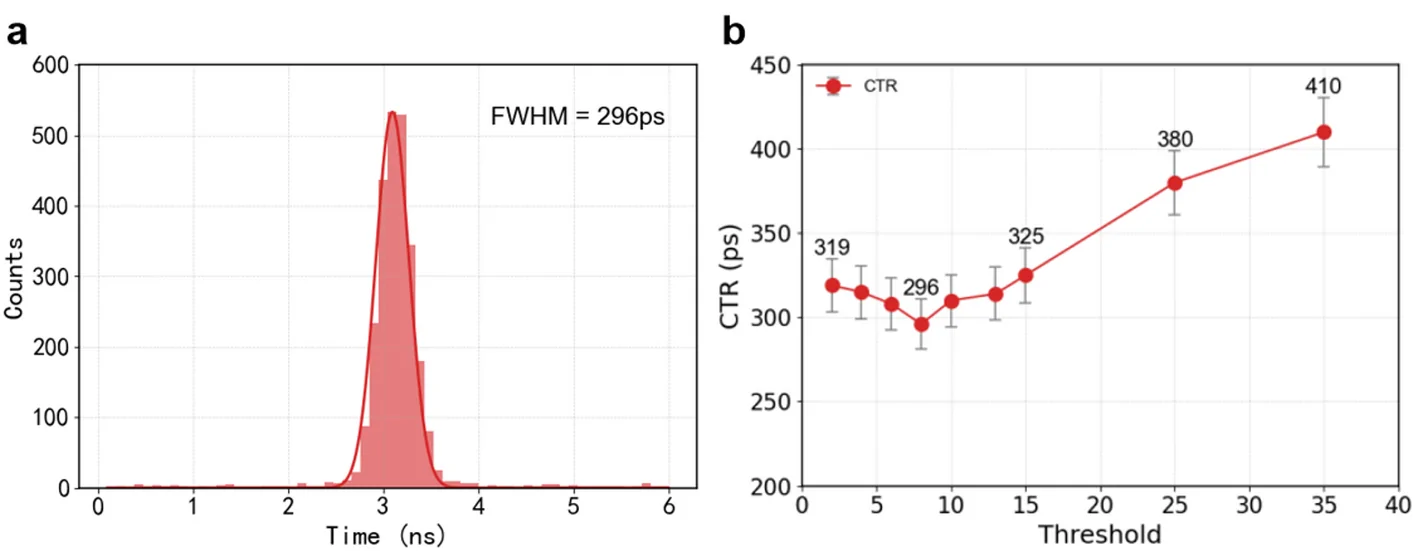
(**a**) Timing spectrum at an ASIC threshold of 8, (**b**) The dependence of timing resolution on the ASIC threshold. The timing resolution is measured by the coincidence of two detectors consisting of a single 2 × 2 × 3 mm³ LYSO crystal read out by a 3 × 3 mm² SiPM

Figure 7a shows the timing spectrum measured at an ASIC threshold of 8. Figure 7b shows the timing resolution for different ASIC thresholds from 2 to 35. As the ASIC threshold increases from 2 to 8, the timing resolution improves gradually and reaches the optimum value of 296 ps FWHM at a threshold of 8. As the ASIC threshold exceeds 8, the timing resolution begins to degrade with the increasing of the threshold. At a threshold of 35, the timing resolution worsens to 410 ps. This result is consistent with that of an independent characterization of the same ASIC, where a coincidence timing resolution (CTR) of 289 ± 6 ps was obtained under similar conditions [53]. This measured CTR corresponds to a single-detector timing resolution (DTR) of approximately 209 ps, assuming equal timing contributions from the two identical detectors. The timing resolution obtained here is nearly the best timing resolution that can be achieved by the MPT2321 ASIC for a PET detector using LYSO crystals. In all subsequent experiments, the reference detector was operated at the same bias voltage of 38 V and an ASIC threshold of 8.

### Flood histogram

**Fig. 8:**
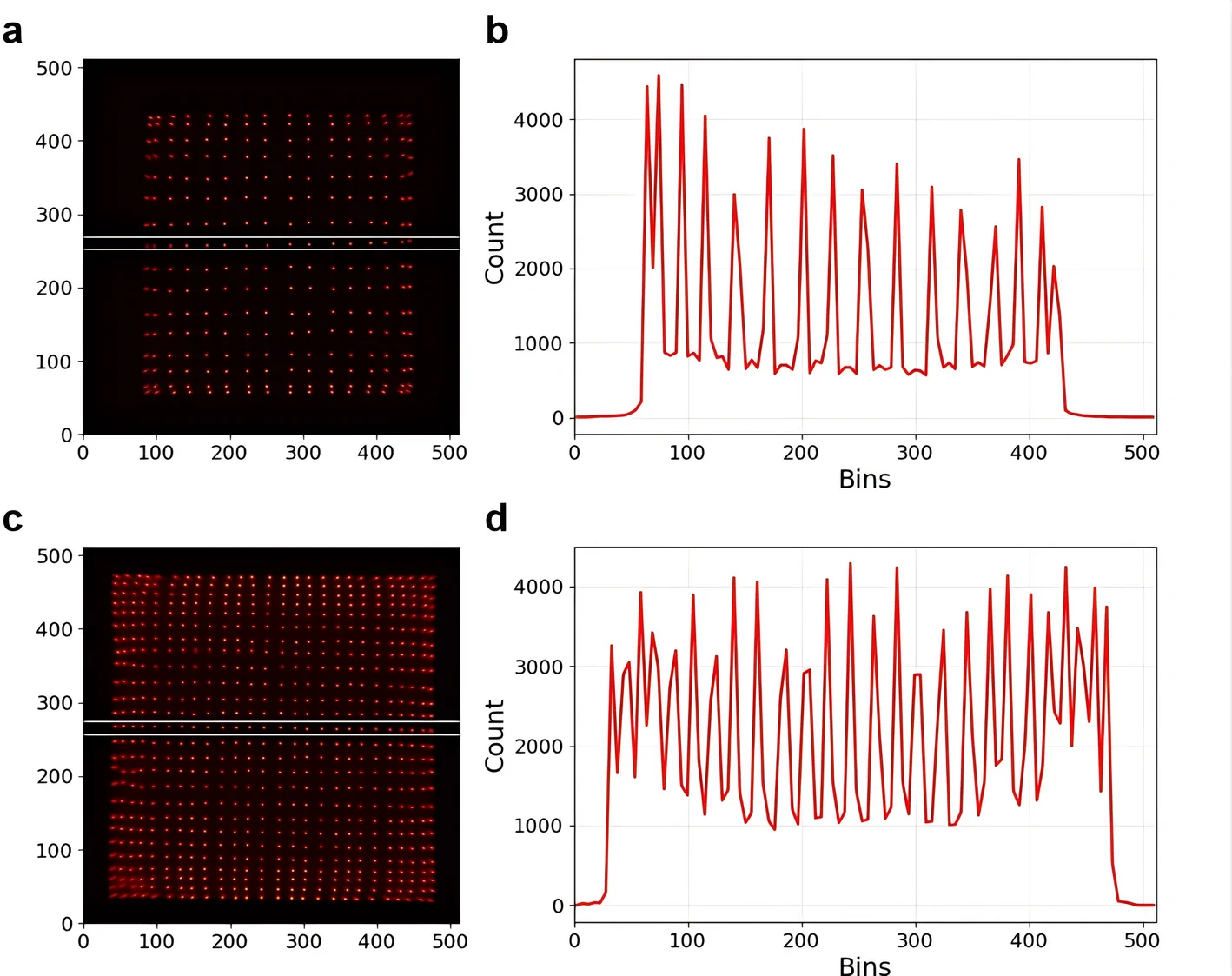
(**a**) Flood histogram, and (**b**) line profile of one middle row of crystals in the 16 × 16 LYSO crystal array detector marked by white lines. (c) Flood histogram, and (d) line profile of one middle row of crystals in the 26 × 26 LYSO crystal array detector marked by white lines

The flood histograms and line profiles of one middle row of the crystals of both detectors are shown in Fig. 8. All crystals are clearly resolved from the flood histograms of both detectors. However, by visual inspection, the crystal identification for the edge crystals is poorer than that of the central crystals for both detectors, and the crystal identification of the 26 × 26 LYSO crystal array is poorer that of the 16 × 16 LYSO crystal array. Average PVRs of 3.59 and 2.61 are achieved from the line profiles of a middle row of the crystals of the 16 × 16 (1.45 × 1.45 × 20 mm³ crystal dimensions) and 26 × 26 (0.75 × 0.75 × 20 mm³ crystal dimensions) LYSO crystal arrays, respectively.

### Energy resolution

**Fig. 9:**
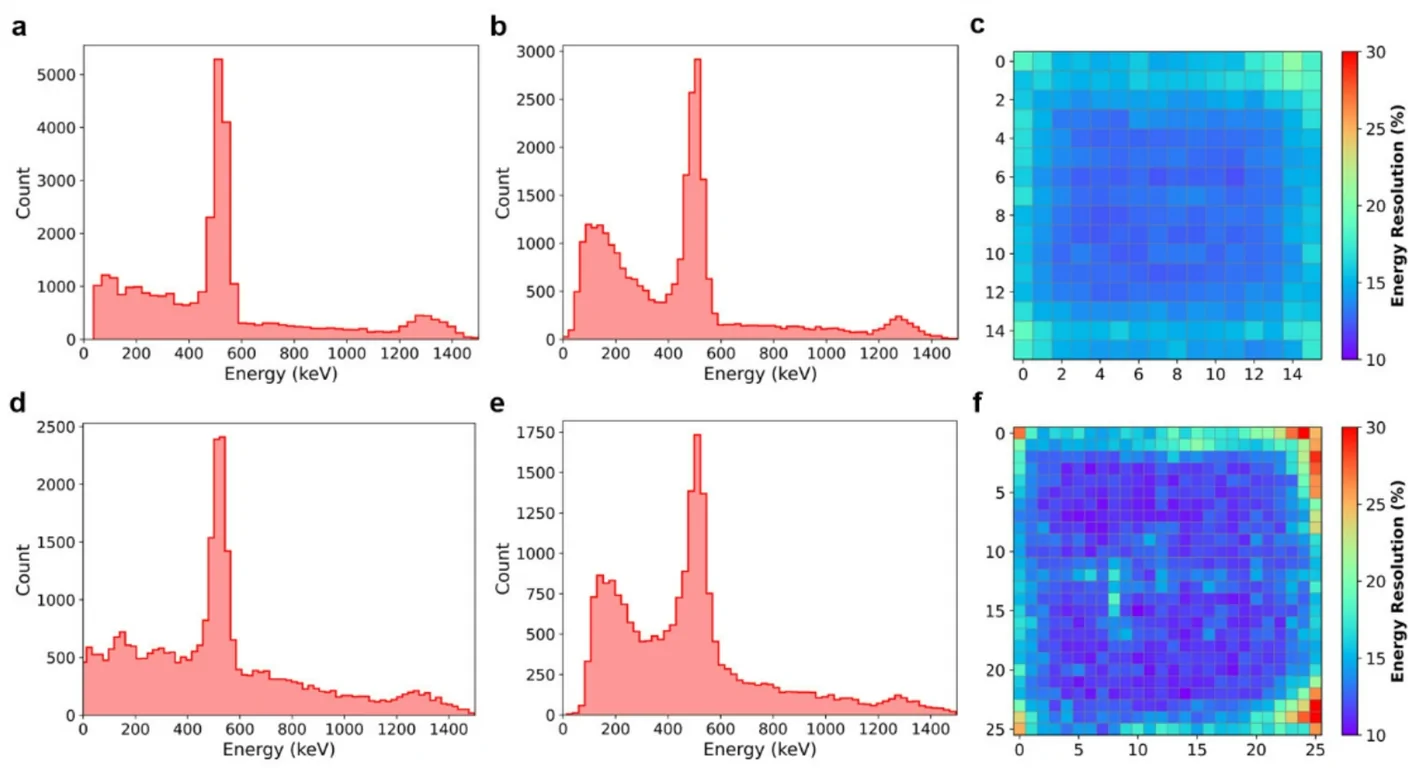
Energy spectra of (**a**) a middle crystal and (**b**) an edge crystal, (**c**) energy resolutions of all individual crystals of the 16 × 16 LYSO crystal array detector. Energy spectra of (**d**) a middle crystal and (**e**) an edge crystal, (f) energy resolutions of all individual crystals of the 26 × 26 LYSO crystal array detector

**Fig. 10:**
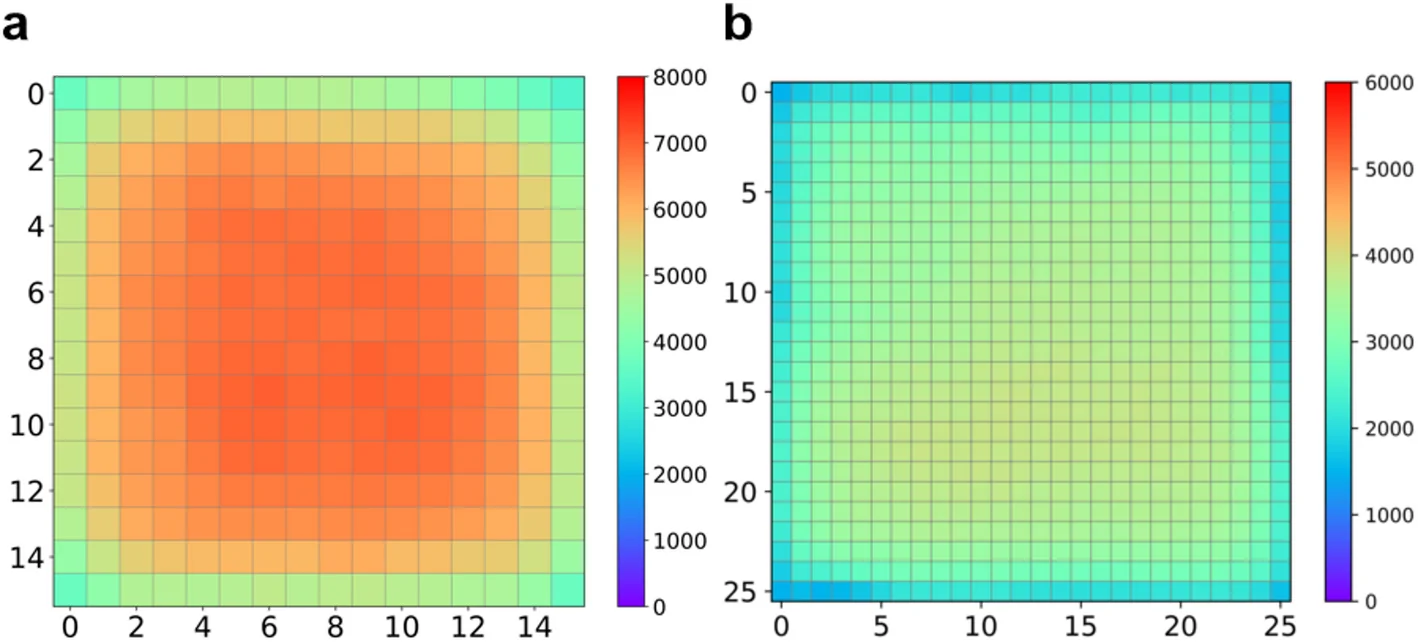
Photopeak positions of all individual crystals of the (**a**) 16 × 16 and (**b**) 26 × 26 LYSO crystal array detectors

Figure 9 presents the energy spectra of a central and an edge crystal, along with the energy resolution of each crystal of the two detectors. The central crystals in both detectors exhibit superior energy resolution compared to the edge crystals. Reducing the crystal width from 1.45 mm to 0.75 mm results in a lower overall energy resolution. For the 16 × 16 LYSO crystal array, an average energy resolution of 14.2 ± 0.4% is achieved, with that of most crystals ranging from 12% to 16%. For the 26 × 26 LYSO crystal array, an average energy resolution of 15.3 ± 0.7% is achieved, with that of most crystals ranging from 12 to 20%. It should be noted that an asymmetry of the energy resolutions of the crystals at the left and right of the 26 × 26 crystal array were observed. This is mainly attributed to a minor misalignment during optical coupling, where the crystal array was slightly shifted from the center of the SiPM array, leading to non-uniform light collection across the detector. The energy resolutions of the detector obtained from the energy spectrum of the detector after the crystal gain equalization are 13.5% and 14.7% for the 16 × 16 and 26 × 26 crystal arrays, respectively. Figure 10 further shows the uncalibrated photopeak positions (ADC values) of all individual crystals of the two detectors. The detector using smaller crystals exhibits lower photopeak positions as compared to the detector using larger crystals.

### DOI resolution

**Fig. 11:**
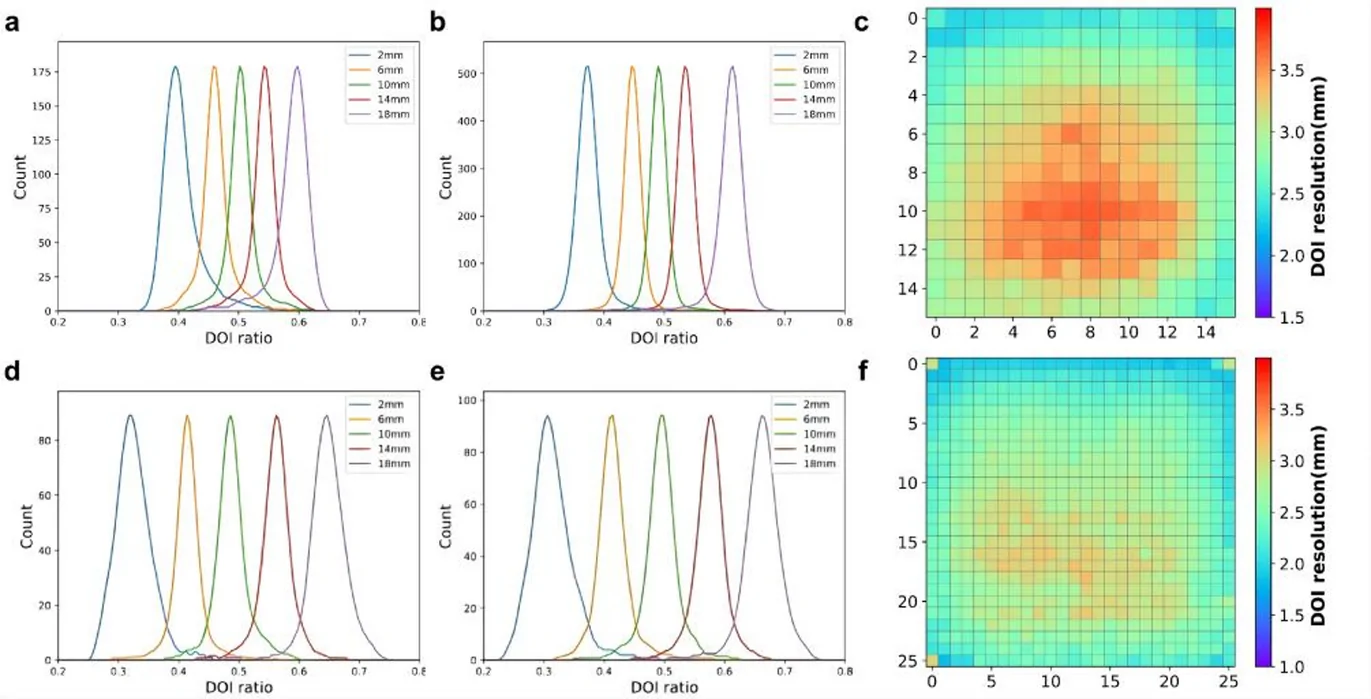
DOI ratio histograms of (**a**) a middle crystal and (**b**) an edge crystal measured at five depths of 2, 6, 10, 14, and 18 mm from one end, (**c**) DOI resolutions averaged over all five depths of all individual crystals of the 16 × 16 LYSO crystal array detector. DOI ratio histograms of (**d**) a middle crystal and (**e**) an edge crystal measured at five depths of 2, 6, 10, 14, and 18 mm from one end, (f) DOI resolution averaged over all five depths of all individual crystals of the 26 × 26 LYSO crystal array detector

Figures 11 presents the DOI ratio histograms of one central and one edge crystal evaluated at depths of 2, 6, 10, 14, and 18 mm, along with the DOI resolutions averaged across the five measurement depths for each crystal of the two detectors. The DOI resolutions of the 26 × 26 LYSO array detector are better than those of the 16 × 16 LYSO array detector, as smaller crystals result in more scintillation photon loss and lead to a larger DOI ratio range. The DOI resolutions of the edge crystals are better than those of the center crystals for both detectors since the DOI ratio ranges of the edge crystals are larger and the inter-crystal scattering events that degrade the DOI resolution have higher probability to be assigned to the center crystals.

The mean DOI resolutions across all crystals and depths are 3.04 ± 0.37 mm and 2.56 ± 0.46 mm for the 16 × 16 and 26 × 26 LYSO array, respectively.

### Timing resolution

**Fig. 12:**
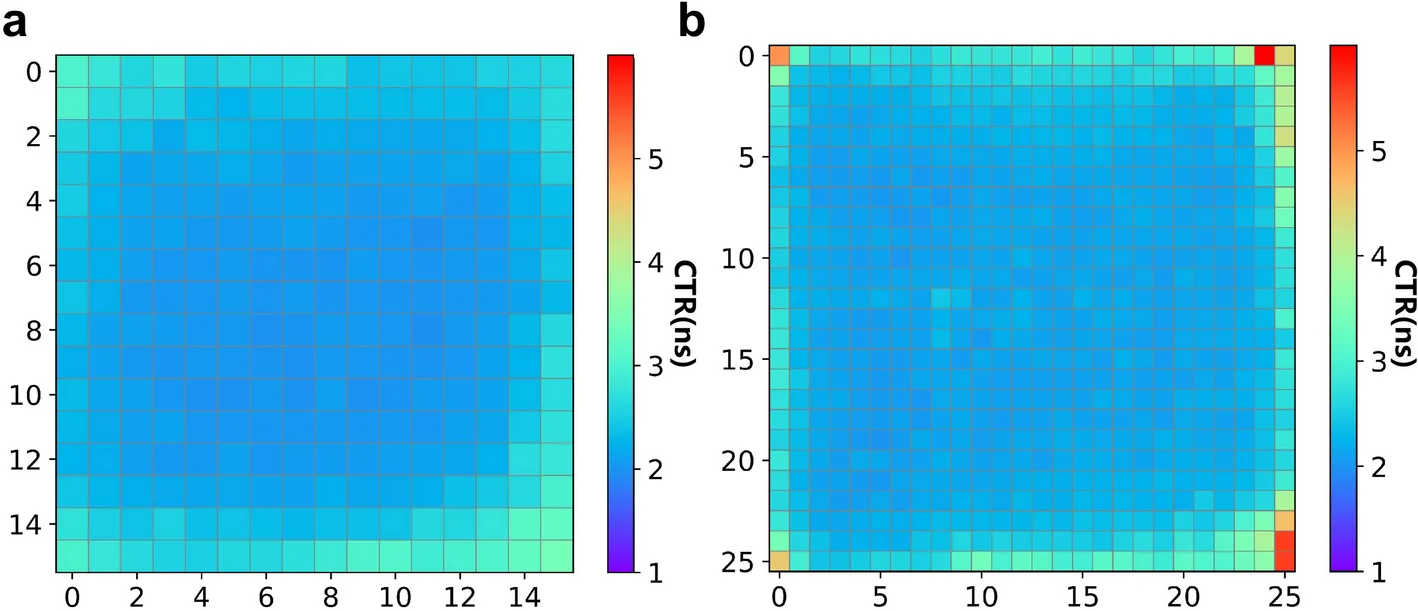
Detector timing resolutions of all individual crystals of the (**a**) 16 × 16 and (**b**) 26 × 26 LYSO crystal array detectors

Figure 12 presents the DTRs for all crystals in both detectors. The DTRs averaged over all crystals are 2.24 ± 0.10 ns and 2.38 ± 0.20 ns for the 16 × 16 and 26 × 26 LYSO arrays, respectively. These DTR values were obtained by quadratically subtracting the contribution of the reference detector (209 ps) from the measured FWHM of the timing spectra.

The central crystals demonstrate better timing performance than the edge crystals, and the detector with a larger crystal size provides better timing performance than the detector with a smaller crystal size, as the SiPMs detect more scintillation photons in both cases.

**Table 2:** Summary of the average energy, DOI and timing resolutions of the two detectors

Detector #	Crystal array	Crystal Dimension (mm³)	Energy resolution (%)	DOI resolution (mm)	DTR (ns)
1	16 ⋅ 16	1.45 × 1.45 × 20	14.2 ± 0.4	3.04 ± 0.37	2.24 ± 0.10
2	26 ⋅ 26	0.75 × 0.75 × 20	15.3 ± 0.7	2.56 ± 0.46	2.38 ± 0.20

## Discussion

This work evaluated two dual-ended PET detectors with high spatial resolution using an electronics scheme that included a row–column multiplexed readout circuit for signal acquisition and dedicated ASICs for signal processing. The results in this study demonstrated that the MPT2321 ASIC, originally designed for the signal processing of individual SiPMs, is well-suited for processing signals from a highly multiplexed SiPM readout circuit. Compared to individual SiPM signal readout electronics currently used in most clinical TOF PET scanners, the electronics scheme evaluated in this work reduces the number of electronics channels by a factor of 16 at the cost of degradation of the timing resolution of the detector from ~ 300 ps to ~ 2 ns. A 2 ns timing resolution is acceptable for a small animal PET scanner. In this work, both the time-walk and DOI-dependent timing corrections were not performed, but only small improvement of the timing resolution is expected from those corrections [56]. The poor timing resolution of this work is mainly limited by the highly multiplexed signal readout scheme.

The MPT2321 ASICs can perform the signal amplification, timing pick-up, timing-to-digital converter and analog-to-digital converter. The data obtained from the ASICs can be directly saved as the singles data or sent to the coincidence processing unit electronics for online coincidence processing. In our previous small animal SIAT aPET scanner consisting of 48 dual-ended readout detectors [12], 12 bulky 32-channel singles processing unit electronics were used. The size of the printed circuit board of a 32-channel singles processing unit is ~ 35⋅50 cm^2^ and the cost is ~ 10k dollars. The cost, complexity, power consumption and required space of the electronics can be much reduced if the MPT2321 ASICs are used since only 12 small ASIC chips are required for the whole scanner. A recently reported S-PET system [57] consists of 20 detector modules using dual-layer scintillators of LYSO and BGO. Each detector module employs two 8 × 8 SiPM arrays read out individually using two 64 channel TOFPET2 ASICs. The S-PET scanner has 2560 readout channels and achieves a coincidence timing resolution of 0.76 ns for LYSO to LYSO, 2.06 ns for LYSO to BGO and 3.17 ns for BGO to BGO. The number of electronics channels can be reduced from 2560 to 160 or 80 if the proposed electronics scheme with 64 to 4 or 128 to 4 signal multiplexing is used. Although the timing resolution of the detectors may more or less degraded, the complexity of the electronics can be much simplified and the cost of the electronics can be much reduced.

Compared with one-to-one crystal-to-photosensor coupling schemes, multiplexed Anger-logic-type readout approaches may exhibit degradation of crystal separation performance at high count rates due to signal pile-up and increased uncertainty in position estimation. In this study, detector characterization was performed under relatively low count-rate conditions typical for PET detector evaluation. Therefore, the dependence of PVR and DOI resolution on gamma flux rate was not systematically investigated. Future work will evaluate the high-rate performance of the proposed multiplexed readout scheme for practical scanner applications.

In this work, only a detector consisting of LYSO crystals of 0.75 × 0.75 × 20 mm³ aimed to develop high performance small animal PET scanner and a detector consisting of LYSO crystals of 1.45 × 1.45 × 20 mm³ mm³ aimed to develop high performance dedicated brain and breast PET scanners were measured. The electronics scheme proposed in this work is highly scalable and can be used to develop different types of PET scanners using a variety number of detectors.

## Conclusion

As summarized in Table 2, the detector using a 16 ⋅ 16 LYSO crystal array with a crystal dimension of 1.45 × 1.45 × 20 mm³ provided a flood histogram with all crystals clearly distinguishable, an energy resolution of 14.2 ± 0.4%, a DOI resolution of 3.04 ± 0.37 mm, and a DTR of 2.24 ± 0.10 ns. The detector using a 26 ⋅ 26 LYSO crystal array with a crystal dimension of 0.75 × 0.75 × 20 mm³ provided a flood histogram with all crystals clearly resolved, an energy resolution of 15.3 ± 0.7%, a DOI resolution of 2.56 ± 0.46 mm and a DTR of 2.38 ± 0.20 ns. The detectors and electronics scheme evaluated in this study can enable the development of low electronics cost and high-performance small-animal and dedicated breast PET scanners with simplified electronics in the future.

## Data Availability

The data that support the findings of this study are available from the corresponding author upon reasonable request.

## Abbreviations

ADC: Analog-to-digital converter
ASIC: Application-specific integrated circuit
CT: Computed tomography
DOI: Depth of interaction
FWHM: Full width at half maximum
LVDS: Low-voltage differential signaling
LYSO: Lutetium–tttrium oxyorthosilicate
MPT2321: MicroParity technology 32-channel ASIC
PVR: Peak-to-valley ratio
PET: Positron emission tomography
PMT: Photomultiplier tube
SiPM: Silicon photomultiplier
TOF: Time-of-flight
